# Clinical features and rare complications in 132 patients with hepatic glycogenosis

**DOI:** 10.1186/s13023-025-03783-4

**Published:** 2025-08-04

**Authors:** Deniz Kor, Fatma Derya Bulut, Burcu Köşeci, Esra Kara, Ezgi Burgaç, İrem Kaplan, Nazmiye Tüzel Gündüz, Halise Neslihan Önenli Mungan

**Affiliations:** 1 Department of Pediatric Metabolism and Nutrition, Çukurova University, Adana, Turkey; 2 Pediatric Metabolism Clinic, Adana City Hospital, Adana, Turkey; 3 Cengiz Gökçek Gynecology and Pediatrics Hospital, Pediatric Metabolism Clinic, Gaziantep, Turkey

**Keywords:** Glycogen storage disease, Liver involvement, Non-hepatic malignancy

## Abstract

**Background:**

Glycogen storage diseases (GSDs) with liver involvement are classified into subtypes—types 0, Ia, and Ib; III, IV, VI, IX, and XIa, XIb, and XIc, depending on the deficient enzyme. Hypoglycemia and hepatomegaly (except type 0) are hallmarks of the disease; however, muscular and renal tubular involvement, dyslipidemia, and osteopenia can occur. The present study was conducted to highlight the clinical differences and characteristics between types, complications, and long-term outcomes in patients with hepatic GSD.

**Materials and Methods:**

The records of 132 patients with hepatic GSD, confirmed through genetic analysis, were retrospectively reviewed.

**Results:**

Of the 132 patients, 55.3% were male. The consanguinity rate was 75, and 53% of the patients had a family history. The age at diagnosis was 34.36 ± 35.1 months. The frequency distribution was as follows: GSD type III (42.4%), Ia (17.4%), IXa (9.1%), Ib (9.1%), IXc (7.6%), VI (6.8%), IXb (4.5%), IV (2.3%), and 0 (0.8%). The most common presenting symptoms were abdominal distention (40.9%), elevated liver transaminases (14.4%), hepatomegaly (13.6%), hypoglycemia (12.1%), family screening (12.1%), growth retardation (4%), and others (3.8%). Hepatomegaly was found in 84.9%, splenomegaly in 20.5%, short stature in 46.2%, underweight in 14.4%, and obesity in 13.5% of the patients. Non-hepatic malignancy was detected in three patients with GSD type III. The twin rate was 6.1%. The rate of short stature was 46.2% at the time of diagnosis, while it was 15.4% in patients who reached adulthood. The number of twin patients was higher than reported in the literature, and structural anomalies such as intestinal duplication cyst, renal artery stenosis, and pulmonary stenosis, which were not previously reported in association with GSD, along with non-hepatic malignancy, were notable findings in our study.

**Conclusions:**

Liver glycogenosis can present distinct and similar clinical, laboratory, and radiological features, challenging differential diagnosis between types. Our study may guide diagnosing and monitoring common GSDs with hepatic involvement.

## Background

Hepatic glycogen storage diseases (GSDs) are a group of inborn errors of metabolism caused by abnormalities in enzymes that catalyze the synthesis or degradation of glycogen [1]. Their incidence is approximately 1 in 10,000 live births worldwide [2]. This group of diseases is caused by various enzyme deficiencies that result in abnormal glycogen synthesis, or glycolysis, typically within muscles and/or liver cells [3, 4]. GSDs with liver involvement are a complex group of disorders, including GSD type 0 (GYS2, MIM# 240,600), Ia (G6PC, MIM# 232,200), Ib (SLC37A4, MIM# 232,220), III (AGL, MIM # 232,400), IV (GBE1, MIM # 232,500), VI (PYGL, MIM # 232,700), IXa (PHKA2, MIM # 306,000), IXb (PHKB, MIM # 261,750), and IXc (PHKG2, MIM # 613,027). All are inherited in an autosomal recessive manner, except for GSD type IXa, which follows an X-linked recessive inheritance pattern [4]. Hypoglycemia and hepatomegaly are the cardinal features of GSDs that affect the liver. GSD type I results from impaired glucose-6-phosphatase activity and is the most severe liver form of GSD from a euglycemic vantage point because the conversion of glucose-6-phosphate to glucose is the final step of glycogenolysis and gluconeogenesis. GSD types 0, III, VI, and IX are ketotic GSD forms and present with less severe hypoglycemia because lactate, amino acids, and glycerol (from fatty acid oxidation) can serve as precursors for gluconeogenesis [5]. Laboratory testing for GSDs should include glucose, electrolytes, liver enzymes, complete blood count, creatine kinase, uric acid, cholesterol, triglycerides, blood gas analysis, ammonia, and lactate, preferably after fasting. In most cases, genetic testing has replaced enzyme assays as the diagnostic method of choice because these tests are difficult to perform and must be performed by an experienced laboratory. Liver biopsy shows hepatocytes bulging with glycogen and a vacuolated appearance; however, this procedure is now rarely performed because genetic testing is readily available and less invasive [6]. Despite the variability in the clinical course among hepatic GSDs, the main goal of treatment is preventing hypoglycemia to avoid hypoglycemic seizures and long-term complications, such as hepatic adenomas, renal dysfunction, and growth impairment [7, 8]. Basic treatment comprises frequent meals, the ingestion of slow-absorption carbohydrates, such as uncooked starch, and dietary restriction of fructose, galactose, sucrose, and lactose, which can aggravate hyperlactacidemia [9, 10]. When dietary control fails or long-term complications persist, liver transplantation should be considered [11, 12].

This paper aims to present a comprehensive case series of hepatic GSD to determine the frequency and differentiation of subtypes and report clinical, laboratory, and radiological findings, long-term follow-up data, complications, and additional issues that have not been previously documented in the literature.

## Methods

### Data sources

This study included 132 patients with GSDs who were diagnosed and followed up at the Department of Pediatric Metabolism and Nutrition, Çukurova University, Adana, Turkey. The diagnosis was confirmed by genetic analysis of all patients. The medical records of the patients were retrospectively reviewed for data, including sex, age at diagnosis, growth parameters, weight and height at diagnosis and the last visit, presenting symptoms, duration of the follow-up period, laboratory analysis results, and family history. Standard deviation (SD) values were calculated using anthropometric references from Turkish children [13]. Detailed laboratory findings were recorded. This retrospective study was approved by the Ethics Committee of Çukurova University Medical Faculty (IRB approval reference number/date:125/16.09.2022). Informed consent was obtained from each patient and their family members according to the ethical standards of the Institutional Ethics Committee (Çukurova University Faculty of Medicine Non-Invasive Clinical Research Ethics Commission) and the Declaration of Helsinki.

### Data analysis

Categorical variables are expressed as numbers and percentages, whereas continuous variables are expressed as means and SDs or medians and minimum–maximum as appropriate. To compare paired variables measured at diagnosis and the last visit, the Wilcoxon signed-rank test was used. All analyses were performed using Statistical Package for the Social Sciences (version 20.0). *P*-values < 0.05 were used to denote statistical significance.

## Results

### General clinical characteristics

**Fig. 1:**
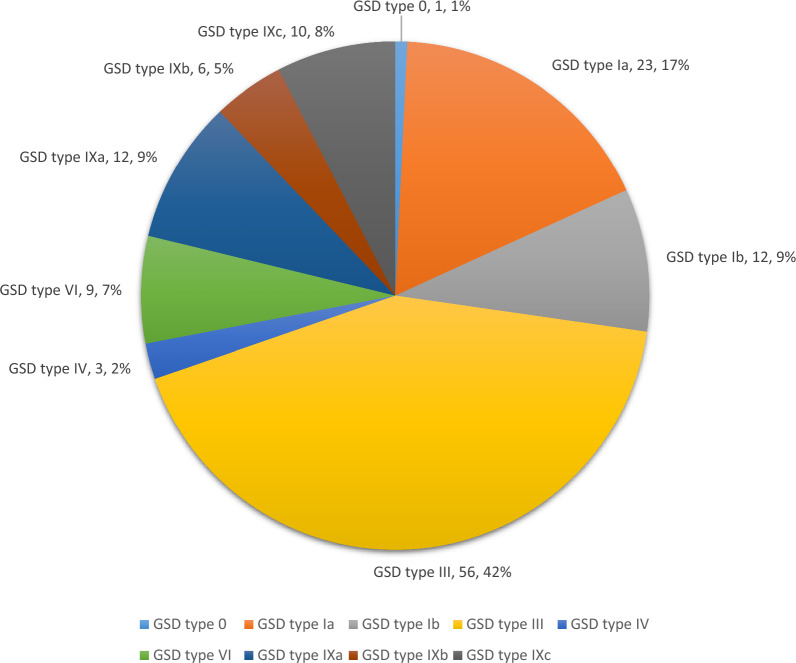
The subtypes of GSD

In this study, 132 patients (59 females and 73 males) with GSDs were included. The GSD subtypes are shown in Fig. 1. In the study group, GSD type III was the most common, accounting for 42.4% (n = 56), type I at 26.5% [35], and type IX at 21.2% [28].

The median age at diagnosis was 34.36 ± 35.1 months (range, 0–177 months). The median follow-up period was 77.35 ± 86.1 months (range, 0–494 months). The earliest diagnosed types were Ia and Ib, while the latest diagnosed types were IXb and VI. The median age at the last follow-up was 111.65 ± 93.0 months (range, 14–586). Twenty-three patients (17.4%) were followed up for > 10 years. At the last admission, 13 patients were aged > 18 years.

**Fig. 2:**
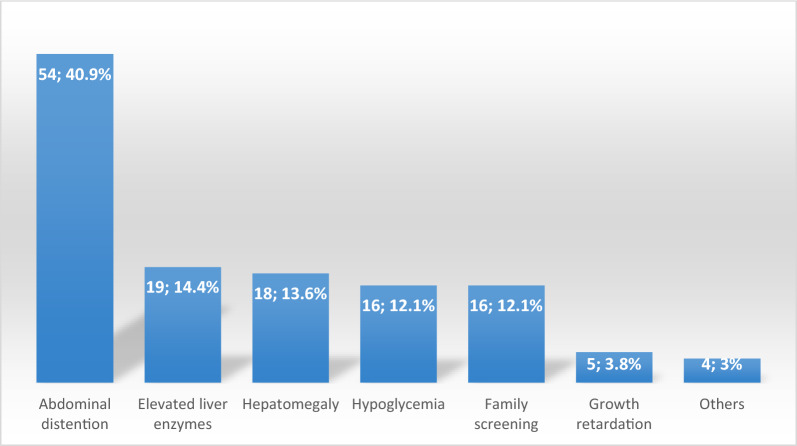
The presenting findings of the patients

The patients' presenting findings are shown in Fig. 2. The frequency of consanguineous marriage among the patients was 75%, and the percentage of patients with a family history of GSD was 53%. Eight of the patients were twins (6.1%). Among the three pairs of twin patients, two were diagnosed with type Ib and one with type IXc GSD. The twins of the other two patients were not affected; of these, one had type Ia GSD and the other had type III GSD.

**Fig. 3:**
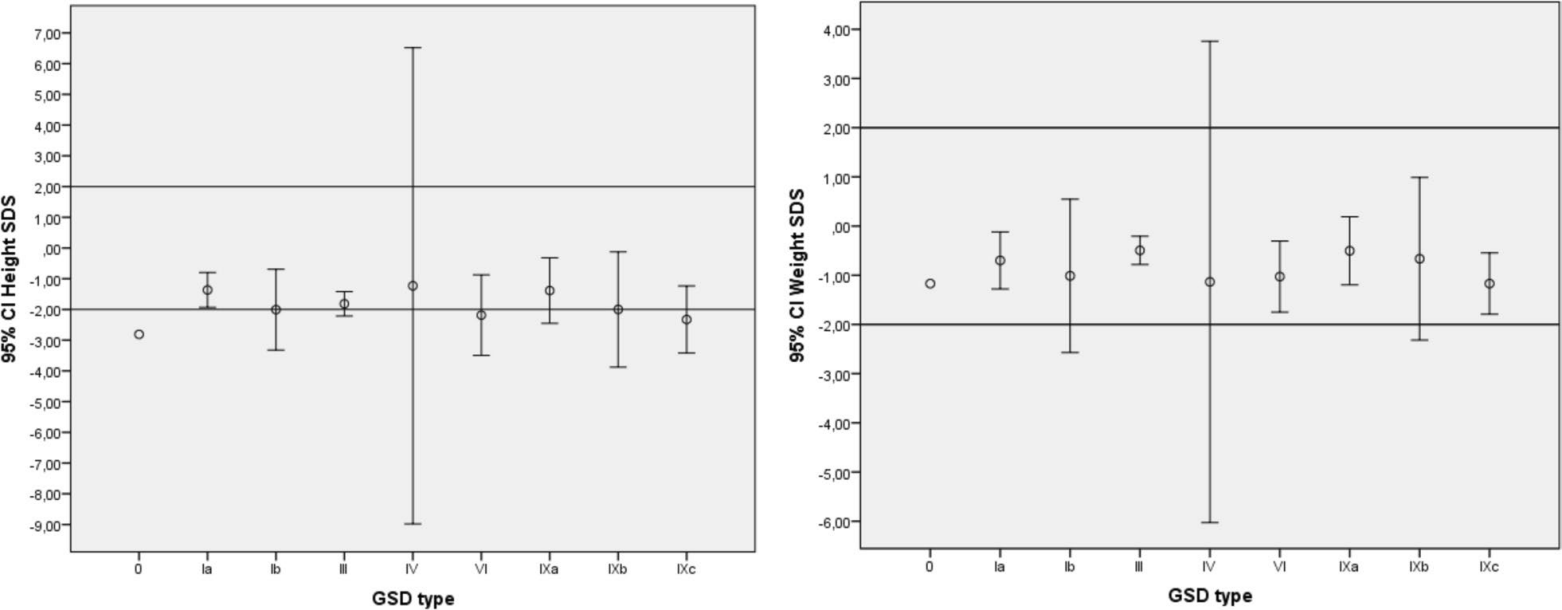
Height and body weight SDS of the patients at the time of diagnosis by GSD type

Physical examination revealed hepatomegaly in 93.9% (124/132) and splenomegaly in 20.5% (27/132) of the cases. In the anthropometric assessment, 46.2% (61/132) of the cases had short stature, 14.4% (19/132) were underweight according to age, and 13.5% (18/132) were obese based on the body mass index (BMI). The demographic characteristics of the patients based on the GSD type are presented in Table 1, and the growth charts are shown in Fig. 3.

**Table 1:** Demographic and clinical characteristics of patients by GSD types

	0a	Ia	Ib	III	IV	VI	IXa	IXb	IXc	Total
Cases (n)	1 (%0.8)	23 (%17.4)	12 (%9.1)	56 (%42.4)	3 (%2.3)	9 (%6.8)	12 (%9.1)	6 (%4.5)	10 (%7.6)	132 (%100)
Male/Female	− /1	9/14	9/3	23/33	2/1	8/1	12/-	4/2	6/4	74/58
Age at diagnosis (month) (min–max)	69	14.09 ± 18.7 (1–82)	21.17 ± 12.6 (7–47)	35.89 ± 36.9 (0–177)	36.33 ± 21.6 (16–59)	60.0 ± 23.8 (33–105)	43.67 ± 41.7 (2–135)	75.5 ± 54.8 (16–129)	25.3 ± 27.4 (3–93)	34.36 ± 35.1 (0–177)
Duration of follow-up (month) (min–max)	44	75.71 ± 60.8 (3–210)	40.89 ± 49.9 (5–161)	99.47 ± 109.3 (3–494)	145.5 ± 191.6	65.88 ± 44.0 (10–281)	43.7 ± 32.3 (23–152)	69.8 ± 55.8 (19–165)	32.63 ± 47.3 (0–145)	77.35 ± 86.1 (0–494)
Current age (month) (min–max)	113	90.33 ± 61.3 (14–215)	65.11 ± 52.9 (16–177)	136.18 ± 115.9 (14–586)	170.5 ± 204.4 (26–315)	126.88 ± 39.7 (69–206)	81.10 ± 48.1 (20–144)	136.8 ± 100.7 (38–295)	62.13 ± 43.9 (29–156)	11.65 ± 93.0 (14–586)
Height at diagnosis (SDS) (min–max)	− 2.81	− 1.36 ± 1.2 (− 4.62/0.7)	− 2.0 ± 1.8 (− 5.06/0.11)	− 1.82 ± 1.4 (− 5.52/0.87)	− 1.23 ± 0.86 (− 1.84/-0.62)	− 2.2 ± 1.7 (− 4.18/0.05)	− 1.39 ± 1.6 (− 4.06/1.62)	− 2.0 ± 1.8 (− 4.4/0.2)	− 2.33 ± 1.5 (− 4.09/0.07)	− 1.79 ± 1.5 (− 5.52/1.62)
Current height (SDS) (min–max)	− 2.07	− 2.74 ± 1.7 (− 6.10/− 0.20)	− 2.33 ± 1.5 (− 4.60/− 0.89)	− 1.78 ± 1.2 (− 3.90/1.09)	1.44	− 1.21 ± 0.8 (− 2.68/0.08)	− 1.22 ± 1.7 (− 3.93/2.3)	− 0.69 ± 1.48 (− 1.91/1.45)	− 1.92 ± 2.02 (− 3.88/2.2)	− 1.86 ± 1.5 (− 6.1/2.3)
Hepatomegaly (n)(%)	–	23 (%100)	12 (%100)	54 (%96.4)	2 (%66.7)	9 (%100)	10 (%83.3)	5 (%83.3)	8 (%80)	124 (%93.9)
Splenomegaly (n)(%)	–	–	2 (%16.7)	22 (%39.3)	1 (%33.3)	–	1 (%8.3)	–	1 (%10)	27 (%20.5)

*SDS*, Standarte deviace score

During the routine follow-up, patients were monitored for hepatic malignancies with abdominal ultrasonography and serum alpha-fetoprotein levels every 6 months. In cases with suspicious findings, abdominal magnetic resonance imaging was performed for further evaluation.

### Monitoring of growth

At the final follow-up, 13 patients were ˃18 years of age. All patients, except for two, had achieved their familial target height. The shortest female was 153 cm, the shortest male was 158.5 cm, the tallest female was 171 cm, and the tallest male was 181 cm. At the time of diagnosis, the height of 71 patients (53.8%) was normal [29 (51.8%) with type III, 15 (65.2%) with type Ia, 7 (58.3%) with type Ib, 6 (50%) with type IXa, 4 each with type VI (44.4%) and IXc (40%), and 3 each with types IV (100%) and IXb (50%)]. At the final follow-up, 67 (59.3%) of 113 patients had normal height. At the final follow-up, the distribution of patients with normal height by type was as follows: 31 (55.4%) with type III, 9 (39.1%) with type Ia, 7 each with types VI (77.7%) and IXa (58.3%), 4 each with types Ib (33.3%) and IXb (66.7%), 3 (30%) with type IXc, and 2 (66.7%) with type IV. The heights of 45 patients (34.1%) were normal at both the initial and final follow-ups: 21 with type III, 7 with type Ia, 4 each with types Ib and type IXa, 3 with type VI, and 2 each with types IXb and IXc.

### Laboratory assessment

At the time of diagnosis, 126 (95.5%) and 112 (84.8%) patients had elevated aspartate transaminase (AST) (15–41 U/L) and alanine transaminase (ALT) (7-35U/L) levels, respectively. Among the 108 patients with final follow-up laboratory findings, 91 (84.3%) had elevated AST levels, and 81 (75%) had elevated ALT levels. Mean AST, ALT, and triglyceride (TG) (< 150 mg/dL) levels according to subtypes are presented in Table 2. Although at a very low rate, there were patients whose AST and ALT levels were normal at the time of diagnosis and the final follow-up. When examining by type, the patients with normal AST levels at the time of diagnosis were those with types Ia (2), Ib (2), IV (1), and IXa (1). Patients with normal ALT were types 0 (1), Ia–b (3–6), IV (1), and IXa–b–c (4–3–2). At the final follow-up, the patients with normal AST levels were those with types 0 (1), Ia-b (4–4), VI (1), IXa-b (4–3), while the patients with normal ALT levels were those with types 0 (1), Ia–b (4–5), III (2), VI (4), and IXa–b–c (5–4–2). All samples for lipid profile were collected in a non-fasting state. Hypertriglyceridemia was present in 81 patients (71.1%). Of these, 29 had a value above 500 (500–1968). Hypertriglyceridemia was present in all type Ia and type Ib patients. Low-density lipoprotein levels were elevated in only one type III patient. The uric acid (2,6–6 mg/dL) level was high enough to require allopurinol treatment in 17 (12.9%) patients (7 [30.4%] type Ia, 6 [50%] type Ib, and 4 [7.1%] type III patients). Neutropenia was present in 9 (75%) type Ib patients. These patients were using granulocyte colony-stimulating factor for neutropenia (Table 3).

**Table 2:** Laboratory characteristics of patients by GSD types

	0a	Ia	Ib	III	IV	VI	IXa	IXb	IXc	Total
Cases (n)	1 (%0.8)	23 (%17.4)	12 (%9.1)	56 (%42.4)	3 (%2.3)	9 (%6.8)	12 (%9.1)	6 (%4.5)	10 (%7.6)	132 (%100)
Serum AST (U/L) (15–41)	40	189.86 ± 164.1 (32–759)	82.64 ± 47.8 (28–156)	446.25 ± 314.7 (92–1426)	213.67 ± 167.2 (21–321)	211.33 ± 276.7 (54–894)	162.00 ± 144.8 (29–408)	52.6 ± 16.07 (36–79)	304.60 ± 257.4 (45–883)	292.70 ± 281.8 (21–1426)
Serum ALT (U/L) (7–35)	16	133.95 ± 176.1 (5–884)	51.18 ± 41.9 (12–160)	354.96 ± 233.4 (55–1261)	123.67 ± 116.5 (17–248)	168.56 ± 231 (42–754)	137.00 ± 130.9 (18–457)	57.00 ± 32.4 (28–101)	198.60 ± 203.1 (18–667)	225.41 ± 224.8 (5–1261)
Serum TG (mg/dL) (< 150)	99	1003.39 ± 583.1 (191–1968)	702.7 ± 512.1 (228–1691)	357.08 ± 297.2 (84–1834)	65.50 ± 23.3 (49–82)	192.75 ± 81.8 (117–330)	240.56 ± 206.2 (43–643)	195.00 ± 137.0 (53–393)	317.50 ± 189.4 (54–630)	449.94 ± 438.0 (43–1968)

*ALT*, Alanine transaminase; *AST*, Aspartate transaminase; *TG*, Triglyceride

**Table 3:** Identified mutations

Number	Gender	Type	Gene	Zygosity	Protein change (Nucleotid change)	Protein change (Nucleotid change)	ACMG
1	F	0	*GYS2*	CH	p.R246* (c.736C > T)	p.G549S (c.1645G > A)*	*Novel
2	F	Ia	*G6PC*	H	p.L335fs (c.1004del)	p.L335fs (c.1004del)	Pathogenic
3	F	Ia		H	p.G270V (c.890G > T)	p.G270V (c.890G > T)	Pathogenic
4	F	Ia		H	p.I198fs (c.592-593del)	p.I198fs (c.592-593del)	Pathogenic
5	M	Ia		H	p.I198fs (c.592-593del)	p.I198fs (c.592-593del)	Pathogenic
6	M	Ia		H	p.R83C (c.247C > T)	p.R83C (c.247C > T)	Pathogenic
7	M	Ia		H	p.R83C (c.247C > T)	p.R83C (c.247C > T)	Pathogenic
8	F	Ia		H	p.R83C (c.247C > T)	p.R83C (c.247C > T)	Pathogenic
9	F	Ia		H	p.R83C (c.247C > T)	p.R83C (c.247C > T)	Pathogenic
10	F	Ia		H	p.R83C (c.247C > T)	p.R83C (c.247C > T)	Pathogenic
11	F	Ia		H	p.R83C (c.247C > T)	p.R83C (c.247C > T)	Pathogenic
12	M	Ia		H	p.R83C (c.247C > T)	p.R83C (c.247C > T)	Pathogenic
13	F	Ia		H	p.R83C (c.247C > T)	p.R83C (c.247C > T)	Pathogenic
14	F	Ia		H	p.R83C (c.247C > T)	p.R83C (c.247C > T)	Pathogenic
15	F	Ia		H	p.R83C (c.247C > T)	p.R83C (c.247C > T)	Pathogenic
16	F	Ia		H	p.R83C (c.247C > T)	p.R83C (c.247C > T)	Pathogenic
17	M	Ia		H	p.R83C (c.247C > T)	p.R83C (c.247C > T)	Pathogenic
18	M	Ia		H	p.R83C (c.247C > T)	p.R83C (c.247C > T)	Pathogenic
19	M	Ia		H	p.R83C (c.247C > T)	p.R83C (c.247C > T)	Pathogenic
20	M	Ia		H	p.R83C (c.247C > T)	p.R83C (c.247C > T)	Pathogenic
21	F	Ia		H	p.W160* (c.480G > A)	p.W160* (c.480G > A)	Pathogenic
22	F	Ia		H	p.W160* (c.480G > A)	p.W160* (c.480G > A)	Pathogenic
23	F	Ia		H	p.W160* (c.480G > A)	p.W160* (c.480G > A)	Pathogenic
24	M	Ia		H	p.W77R (c.229 T > C)	p.W77R (c.229 T > C)	Pathogenic
25	M	Ib	*SLC37A4*	H	p.G339D (c.1016G > A)	p.G339D (c.1016G > A)	Pathogenic
26	F	Ib		H	p.G339D (c.1016G > A)	p.G339D (c.1016G > A)	Pathogenic
27	M	Ib		H	p.G339D (c.1016G > A)	p.G339D (c.1016G > A)	Pathogenic
28	F	Ib		H	p.L348fs (c.1042_1043delCT)	p.L348fs (c.1042_1043delCT)	Pathogenic
29	M	Ib		CH	p.L348fs (c.1042_1043delCT)	c.444delTinsCCA*	*Novel
30	M	Ib		CH	p.L348fs (c.1042_1043delCT)	c.444delTinsCCA*	*Novel
31	M	Ib		H	p.L348fs (c.1042_1043delCT)	p.L348fs (c.1042_1043delCT)	Pathogenic
32	M	Ib		H	p.L348fs (c.1042_1043delCT)	p.L348fs (c.1042_1043delCT)	Pathogenic
33	F	Ib		H	p.G122E (c.365G > A)	p.G122E (c.365G > A)	Likely pathogenic
34	M	Ib		H	p.R300C (c.898C > T)	p.R300C (c.898C > T)	Pathogenic
35	M	Ib		H	p.R415* (c.1243C > T)	p.R415* (c.1243C > T)	Pathogenic
36	M	Ib		H	p.W128R (c.382 T > C)	p.W128R (c.382 T > C)	VUS
37	F	III	*AGL*	H	3–4. exon deletion	3–4. exon deletion	Novel
38	F	III		H	3–4. exon deletion	3–4. exon deletion	Novel
39	M	III		H	p.R864* (c.2590C > T)	p.R864* (c.2590C > T)	Pathogenic
40	F	III		H	c.2950-2A > G	c.2950-2A > G	Pathogenic
41	M	III		H	p.T1242fs (c.3772-3776del)	p.T1242fs (c.3772-3776del)	Pathogenic
42	M	III		H	c.3837-1G > A	c.3837-1G > A	Pathogenic
43	M	III		H	IVS5 + 3A > G (c.664 + 3A > G)	IVS5 + 3A > G (c.664 + 3A > G)	Pathogenic
44	F	III		H	p.E1326X	p.E1326X	VUS
45	F	III		H	IVS13-11A > G (c.1736-11A > G)	IVS13-11A > G (c.1736-11A > G)	Likely pathogenic
46	F	III		H	IVS13-11A > G (c.1736-11A > G)	IVS13-11A > G (c.1736-11A > G)	Likely pathogenic
47	M	III		H	IVS31-12A > G (c.4260-12A > G)	IVS31-12A > G (c.4260-12A > G)	Pathogenic
48	F	III		H	IVS5 + 3A > G (c.664 + 3A > G)	IVS5 + 3A > G (c.664 + 3A > G)	Pathogenic
49	M	III		H	p.721-722delYVinsNG (c.2161-2165delTATGT)	p.721-722delYVinsNG (c.2161-2165delTATGT)	Novel
50	F	III		H	p.D251fs (c.753-756del)	p.D251fs (c.753-756del)	Pathogenic
51	F	III		H	p.D251fs (c.753-756del)	p.D251fs (c.753-756del)	Pathogenic
52	F	III		H	p.D501fs (c.1497_1500dup)	p.D501fs (c.1497_1500dup)	Pathogenic
53	M	III		H	p.E340fs (c.1020del)	p.E340fs (c.1020del)	Pathogenic
54	F	III		CH	p.L147P (c.440 T > C)	IVS7 + 1G > A	Novel
55	M	III		H	p.L147P (c.440 T > C)	p.L147P (c.440 T > C)	Novel
56	F	III		H	p.L168fs (c.500dup)	p.L168fs (c.500dup)	Pathogenic
57	F	III		H	p.L381*(c.1141_1141delT)	p.L381*(c.1141_1141delT)	Novel
58	F	III		H	p.L381*(c.1141_1141delT)	p.L381*(c.1141_1141delT)	Novel
59	F	III		H	p.Q1376* (c.4126C > T)	p.Q1376* (c.4126C > T)	Pathogenic
60	M	III		H	p.Q1376* (c.4126C > T)	p.Q1376* (c.4126C > T)	Pathogenic
61	F	III		H	p.Q1376* (c.4126C > T)	p.Q1376* (c.4126C > T)	Pathogenic
62	F	III		H	p.Q1376* (c.4126C > T)	p.Q1376* (c.4126C > T)	Pathogenic
63	M	III		H	p.Q1376* (c.4126C > T)	p.Q1376* (c.4126C > T)	Pathogenic
64	F	III		H	p.Q1376* (c.4126C > T)	p.Q1376* (c.4126C > T)	Pathogenic
65	M	III		H	p.Q1376* (c.4126C > T)	p.Q1376* (c.4126C > T)	Pathogenic
66	F	III		H	p.Q1376* (c.4126C > T)	p.Q1376* (c.4126C > T)	Pathogenic
67	M	III		H	p.Q1376* (c.4126C > T)	p.Q1376* (c.4126C > T)	Pathogenic
68	F	III		H	p.Q1376* (c.4126C > T)	p.Q1376* (c.4126C > T)	Pathogenic
69	F	III		H	p.Q1376* (c.4126C > T)	p.Q1376* (c.4126C > T)	Pathogenic
70	M	III		H	p.Q1376* (c.4126C > T)	p.Q1376* (c.4126C > T)	Pathogenic
71	M	III		H	p.Q1376* (c.4126C > T)	p.Q1376* (c.4126C > T)	Pathogenic
72	M	III		H	p.Q1376* (c.4126C > T)	p.Q1376* (c.4126C > T)	Pathogenic
73	F	III		H	p.Q1376* (c.4126C > T)	p.Q1376* (c.4126C > T)	Pathogenic
74	F	III		H	p.Q1376* (c.4126C > T)	p.Q1376* (c.4126C > T)	Pathogenic
75	F	III		CH	p.Q1376* (c.4126C > T)	c.958 + 1G > A	Pathogenic
76	M	III		H	p.Q667* (c.1999C > T)	p.Q667* (c.1999C > T)	Novel
77	M	III		H	p.Q667* (c.1999C > T)	p.Q667* (c.1999C > T)	Novel
78	M	III		H	p.Q937* (c.2809C > T)	p.Q937* (c.2809C > T)	Novel
79	F	III		H	p.Q667* (c.1999C > T)	p.Q667* (c.1999C > T)	Novel
80	F	III		H	p.R1101G(c.3301A > G)	p.R1101G(c.3301A > G)	Novel
81	F	III		H	p.R343W (c.1027C > T)	p.R343W (c.1027C > T)	Conflicting classifications of pathogenicity
82	M	III		H	p.R343W (c.1027C > T)	p.R343W (c.1027C > T)	Conflicting classifications of pathogenicity
83	M	III		H	p.R343W (c.1027C > T)	p.R343W (c.1027C > T)	Conflicting classifications of pathogenicity
84	F	III		H	p.R343W (c.1027C > T)	p.R343W (c.1027C > T)	Conflicting classifications of pathogenicity
85	F	III		H	p.R595* (c.1783C > T)	p.R595* (c.1783C > T)	Pathogenic
86	F	III		H	p.R864* (c.2590C > T)	p.R864* (c.2590C > T)	Pathogenic
87	M	III		H	p.R864* (c.2590C > T)	p.R864* (c.2590C > T)	Pathogenic
88	M	III		H	p.R864* (c.2590C > T)	p.R864* (c.2590C > T)	Pathogenic
89	F	III		CH	p.R910*(c.2728C > T)	p.Q1376* (c.4126C > T)	Pathogenic
90	F	III		H	p.S757fs*18 (c.2270_2273delCATT)	p.S757fs*18 (c.2270_2273delCATT)	Pathogenic
91	M	III		H	p.T1258fs* (c.3772_3776del)	p.T1258fs (c.3772_3776del)	Pathogenic
92	F	III		H	p.W1099* (c.3297G > A)	p.W1099* (c.3297G > A)	Pathogenic
93	F	IV	*GBE1*	H	p.K521E (c.1561A > G)	p.K521E (c.1561A > G)	VUS
94	M	IV		H	p.K521E (c.1561A > G)	p.K521E (c.1561A > G)	VUS
95	M	IV		H	p.K521E (c.1561A > G)	p.K521E (c.1561A > G)	VUS
96	M	VI	*PYGL*	H	p.N377K (c.1131C > G)	p.N377K (c.1131C > G)	Pathogenic
97	M	VI		H	c.1475G > T (p.W492L)	c.1475G > T (p.W492L)	Novel
98	M	VI		H	c.1475G > T (p.W492L)	c.1475G > T (p.W492L)	Novel
99	M	VI		H	IVS14 + 1G > T (c.1768 + 1G > T)	IVS14 + 1G > T (c.1768 + 1G > T)	Pathogenic
100	M	VI		H	IVS14 + 1G > T (c.1768 + 1G > T)	IVS14 + 1G > T (c.1768 + 1G > T)	Pathogenic
101	F	VI		H	IVS15-2A > C (c.1828-2A > C)	IVS15-2A > C (c.1828-2A > C)	Novel
102	M	VI		H	IVS15-2A > C (c.1828-2A > C)	IVS15-2A > C (c.1828-2A > C)	Novel
103	M	VI		H	p.I71IVfs*9 (c.2127-2130dup)	p.I71IVfs*9 (c.2127-2130dup)	Novel
104	M	VI		H	p.M1K (c.2 T > A)	p.M1K (c.2 T > A)	VUS
105	M	IXa	*PHKA2*	Hem	IVS23 + 1G > A (c.2597 + 1G > A)		Likely pathogenic
106	M	IXa		Hem	IVS23 + 1G > A (c.2597 + 1G > A)		Likely pathogenic
107	M	IXa		Hem	IVS23 + 1G > A (c.2597 + 1G > A)		Likely pathogenic
108	M	IXa		Hem	p.A1076S (c.3226G > T)		Novel
109	M	IXa		Hem	p.A1076S (c.3226G > T)		Novel
110	M	IXa		Hem	p.K253N (c.759A > C)		Novel
111	M	IXa		Hem	p.Q474* (c.1420C > T)		Pathogenic
112	M	IXa		Hem	p.R295C (c.883C > T)		Pathogenic
113	M	IXa		Hem	p.R295C (c.883C > T)		Pathogenic
114	M	IXa		Hem	p.R295C (c.883C > T)		Pathogenic
115	M	IXa		Hem	p.R45W(c.133C > T)		Pathogenic
116	M	IXa		Hem	p.S405Pfs*22 (c.1212_1213del)		Novel
117	F	IXb	*PHKB*	CH	p.Y763C (c.2309A > G)	p.S314N (c.941G > A)	Likely benign
118	M	IXb		H	p.L782fs*2 (c.2344_2345delTT)	p.L782fs*2 (c.2344_2345delTT)	Novel
119	M	IXb		H	p.L38H (c.113 T > A)	p.L38H (c.113 T > A)	Novel
120	M	IXb		H	p.Q657K (c.1969C > A)	p.Q657K (c.1969C > A)	Conflicting classifications of pathogenicity
121	M	IXb		H	p.Q657* (c.1969C > T)	p.Q657* (c.1969C > T)	Pathogenic
122	F	IXb		H	p.Q657* (c.1969C > T)	p.Q657* (c.1969C > T)	Pathogenic
123	F	IXc	*PHKG2*	H	p.H145fs*10 (c.433delC)	p.H145fs*10 (c.433delC)	Pathogenic
124	M	IXc		H	p.D28* (c.77_78insTAAA)	p.D28* (c.77_78insTAAA)	Novel
125	M	IXc		H	p.D28* (c.77_78insTAAA)	p.D28* (c.77_78insTAAA)	Novel
126	M	IXc		H	p.E157K (c.469G > A)	p.E157K (c.469G > A)	Pathogenic
127	F	IXc		H	p.L267L (c.801G > A)	p.L267L (c.801G > A)	Novel
128	M	IXc		H	p.L267L (c.801G > A)	p.L267L (c.801G > A)	Novel
129	F	IXc		H	p.L267L (c.801G > A)	p.L267L (c.801G > A)	Novel
130	F	IXc		H	p.L267L (c.801G > A)	p.L267L (c.801G > A)	Novel
131	M	IXc		H	p.R76Q (c.227G > A)	p.R76Q (c.227G > A)	VUS
132	M	IXc		H	p.R76Q (c.227G > A)	p.R76Q (c.227G > A)	VUS

*M* Male, *F* Female, *H* Homozygous, *Hem* Hemizygous, *CH* Compound heterozygous, *ACMG* American college of medical genetics; *VUS* Variant of uncertain significance

### Radiological evaluation

Hepatomegaly was present in 93.9% of the patients. Twenty-seven patients (20.5%) had splenomegaly accompanying hepatomegaly. Splenomegaly was detected in 22 patients (39.3%) with type III, 2 (16.7%) with type Ib, and one patient each with types IV (33.3%), IXa (8.3%), and IXc (10%). Eight patients without hepatomegaly (6.1%) included two type III, one type IV, two type IXa, one type IXb, and two type IXc patients. Among the patients with normal ultrasound findings, five had elevated ALT levels, while all had elevated AST levels. Isolated hepatomegaly was present in 20 patients, while in the remaining patients, hepatomegaly was accompanied by increased echogenicity in the parenchyma.

### Liver biopsy

Liver biopsy had been performed on 44 patients before diagnosis. Among these, 10 patients (22.7%) had varying degrees of fibrosis, 6 patients (13.6%) had cirrhosis, and the others had findings consistent with the disease. When examined by type, cirrhosis was found in three patients with type III, two with type IV, and one with type IXc. Biopsy findings consistent with fibrosis were present in six patients with type III, two with type VI, and one with types IXc and Ib.

### Additional issues

Intrauterine growth retardation was present in 16 patients. Calcification of the aorta was present in one patient with type Ia, and pulmonary stenosis was observed in another patient with type Ia. Nephrolithiasis was present in five patients (two with type Ib and one each with types Ia, IV, and III). Additionally, one patient had chronic kidney failure (type Ib), one had focal segmental glomerulosclerosis (type Ib), and one had renal artery stenosis (type Ia). One patient had experienced thromboembolism (type Ia), and another had developed esophageal varices (type III). One patient had a duplication cyst (type Ia), one had pectus carinatum (type VI), one had kyphoscoliosis (type III), and one had a horseshoe kidney (type IV) as structural anomalies. Cholelithiasis was detected in two patients, one with type Ib and the other with type IX. Familial Mediterranean fever was present in two patients (types III and VI) and hemophilia B in two patients (both type III). Additionally, one patient had mucopolysaccharidosis type 3 (type Ia), and another had familial hypertriglyceridemia (type IXb).

### Long-term follow-up

Two patients underwent liver transplantation during follow-up. One was a type IXc patient, and the other was a type III patient. The type IXc patient, who was transplanted due to cirrhosis, unfortunately passed away after the transplant. Despite having received an early diagnosis and treatment due to his brother's condition, this patient exhibited a more aggressive course compared to his brother. The other patient was a type III patient who had lost her siblings due to the combination of GSD and acute lymphoblastic leukemia (ALL). After the liver transplantation, she developed polyneuropathy. But she gave birth to a healthy baby without any perinatal complications. Another patient with type III GSD was lost during follow-up due to ALL. One patient with GSD type III received treatment for non-Hodgkin lymphoma, while another patient received treatment for chronic myeloid leukemia. All patients with hemato-oncologic problems were type III patients. No adenomas or hepatocellular carcinoma were observed in any of the patients.

## Discussion

The overall incidence of GSDs is approximately 1 case per 20,000–43,000 live births, and 80% of hepatic GSDs are caused by types I, III, and IX [14]. The main clinical findings of GSDs are hypoglycemia, hepatomegaly, and short stature. It is very difficult to clinically differentiate between the subtypes. The definitive diagnosis and subtype differentiation in patients suspected of having GSD is made through molecular analysis. In addition to severe forms, patients may also present with mild clinical findings. Long-term complications include hepatic lesions such as hepatic adenoma and hepatocellular carcinoma, splenomegaly, renal involvement findings such as chronic kidney disease, chronic kidney failure, urolithiasis, and nephrocalcinosis, hypertension, neurological involvement findings such as cognitive delay, epilepsy, and neuropathy, cardiac findings including cardiomyopathy, QT prolongation, and pulmonary hypertension, findings secondary to cirrhosis including portal hypertension, ascites, and esophageal varices, polycystic ovary syndrome, delayed puberty, osteopenia/osteoporosis, gout, pancreatitis, cholelithiasis, and myeloid malignancy [14].

According to the literature, studies conducted in different regions report that the most commonly observed types are Ia, III, and IXa [2, 15–17]. A study conducted in Turkey involving 38 patients with hepatic GSD reported that the most common type was GSD type III at a rate of 39.5%, followed by type Ia at 31.5% [18]. In the present study, the most common type was GSD type III at 42.4%, followed by type Ia at 17.4% and types IXa and Ib at 9.1%. Additionally, the frequently observed types in the literature, namely types I, III, and IX, constituted 90.2% of the patients in our study. However, the reason for the most commonly observed type not being the same in different studies may be attributed to variations in patient numbers and genotypic differences. In a study evaluating the subgroups of GSD type I, the Ib/Ia ratio was found to be 24% [10]. In a study evaluating GSD type I patients from Turkey, this ratio was found to be 11.1% [19]. In the present study, the Ib/Ia ratio was 52%, and a larger number of patients were diagnosed with Ib. This situation may be attributed to the high frequency of consanguineous marriages and high birth rates in our region. The most common presenting complaints in our patients were abdominal distension, hepatomegaly detected during hospital visits for other reasons, abnormal liver function tests, hypoglycemia, family screening, and growth retardation. Patients with GSD type Ia–b most commonly presented with hypoglycemia, while those with type IXc presented with hepatomegaly, and patients with types III and IXa presented with abdominal distension. Based on the findings of the patients, it can be stated that the presence of abdominal distension upon presentation or the detection of hepatomegaly and/or abnormal liver function tests during hospital visits can serve as an indicative finding for GSD. In a multicenter study involving 231 patients with GSD type Ia, the average age at presentation was reported to be 6 months (ranging from 1 day to 12 years), with 80% of patients presenting before age 1. The most common reasons for presentation were abdominal enlargement in 83% of cases and acute metabolic decompensation in 71% [10]. It was reported that the average age at presentation for patients with GSD type Ib was 4 months (ranging from 1 day to 4 years), with 90% of patients presenting with symptoms before age 1 [10]. In a study conducted in China on patients with hepatic GSDs, the main complaints were reported as abdominal distension or hepatomegaly (20/49), abdominal distension and elevated liver transaminase levels (16/49), hepatosplenomegaly (3/49), abnormal liver function detected during hospital visits for other reasons (6/49), and growth retardation (1/49). It was reported that 91.67% (22/24) of GSD Ia patients had abdominal distension with hepatomegaly. In the same study, the age at diagnosis was reported to be 4.84 ± 3.16 years for type Ia, 2.22 ± 0.86 years for type IIIa, and 2.61 ± 1.19 years for type IXa [20]. In a study examining GSD type VI patients, the median age at diagnosis for 63 cases was found to be 5.3 years [21]. Although the ages at diagnosis vary across different series, in the present study, the earliest diagnosed types were types Ia, Ib, and IXc. Although the mean age at diagnosis was 14.09 ± 18.7 (1–82) months for type Ia patients, the median age at diagnosis was 6 months. Furthermore, since the mean age at diagnosis seemed to be high, the median age at diagnosis was similar to that reported in most studies, and patients received a diagnosis on average within 3 months after their first presentation. While there are some similarities between our results and the data in the literature, the differences are most likely due to environmental factors and genetic differences. The early diagnosis ages of our patients with types Ia and Ib may be attributed to the more pronounced hypoglycemia in this group, which tends to manifest earlier as a clinical symptom.

Interestingly, 6.1% of our patients were twins. According to 2023 data from Turkey, the national rate of multiple births is 3.3%, suggesting a higher-than-expected frequency of twinning in our cohort [22]. Two families with type Ib and one family with type IXc had twin children, and there was also one patient, each with types Ia and III, who had a healthy twin. In other words, 5 out of the 35 patients with types Ia and Ib were twins (14.3%). In the existing literature, a study evaluating 29 patients with GSD type Ia and 7 patients with type Ib reported that 4 of the type Ia patients were 2 twin siblings, and other studies reported Korean twins with GSD Ia and Polish twins with type IIIb [23–25]. The high number of twins in our patient group was a remarkable finding.

In the study conducted by Jorge et al. on 11 patients with GSD type I, it was reported that 8 patients (72.7%) had a normal BMI for their age, while 3 were classified as overweight. When examining height, it was observed that 4 patients (36.4%) were classified as short for their age [26]. Hijazi et al. reported that 40% of patients with GSD type III had a normal BMI, 20% were overweight, and 40% were obese, and the mean BMI of the patients was found to be 28.6 ± 7.4 [27]. In a study that performed phenotyping of GSD type IX, it was reported that growth retardation was observed in 58.3% of patients with GSD type IXa, 87.5% of patients with GSD type IXb, and 70.8% of patients with GSD type IXc [28]. In the present study, when examining the weight SD score (SDS) of the patients at the time of diagnosis, it was found that 19 patients (14.4%) had a body weight of < − 2 SDS (4 type Ia, 4 type Ib, 5 type III, 1 type IV, 1 type VI, 1 type IXa, 1 type IXb, and 2 type IXc). When examining the height SDS, it was found that 61 patients (46.2%) had a height of < 2 SDS, including 1 patient (100%) with type 0, 8 patients (34.8%) with type Ia, 5 patients (41.7%) with type Ib, 27 patients (48.2%) with type III, 5 patients (55.6%) with type IV, 6 patients (50%) with type IXa, 3 patients (50%) with type IXb, and 6 patients (60%) with type IXc.

Due to the prominence of short stature over body weight, when evaluating the BMI SDS in these patients, it was found that only 3 patients had a BMI of < –2 SDS (1 with type Ia, 1 with type III, and 1 with type IV), while 18 patients had a BMI of > + 2 SDS (2 with type Ia, 2 with type Ib, 8 with type III, 1 with type VI, 1 with type IXa, 2 with type IXb, and 2 with type IXc), and 111 patients had normal values. These results emphasize the necessity of using BMI SDS to evaluate the nutritional status of patients with short stature.

In a study conducted on hepatic GSD, 18% of patients were found to have height < − 2 SDS. However, this rate decreased to 7% during follow-up [29]. When 113 patients with anthropometric measurements during follow-up were evaluated, it was found that 46 patients (%40.7) had short stature, 15 patients (%13.3) had body weight < − 2 SDS, and when assessed according to BMI, only 2 patients (types III–IV) were < − 2 SDS, while 16 patients (2 type Ia, 3 type Ib, and 11 type III) were ˃ + 2 SDS. In other words, the rate of short stature had decreased from 46.2% at the time of diagnosis to 40.7% at the final follow-up. Although patients had short stature at the time of diagnosis, its rate among those under long-term follow-up and who reached adulthood was found to be %15.4 (2/13). This indicates that even if patients grow slowly, they are likely to reach their familial target height. The two patients who did not reach their familial target height were types Ia and IXb. Although over 18 years old, the type Ia patient still had open epiphysis in the left wrist X-ray (consistent with a 15-year-old) and continued to experience growth. Short stature is a prominent finding in GSD patients during the early stages; however, through our patients, it was observed that there is a high potential for reaching familial target height at later ages. In general, the findings of our study align with the data in the literature, while the differences may stem from regional genetic variations, dietary factors, and variations in treatment approaches. Regional dietary differences can influence the quality and quantity of the foods consumed by patients, leading to variations in BMI, weight, and height SDS. For hepatic forms of GSDs, parameters such as uric acid, cholesterol, TG, AST, and ALT are used to monitor the disease [30]. As a guideline, in GSD type I, the serum concentrations of AST and ALT increase and often return to normal or near-normal levels with appropriate treatment. In contrast, AST and ALT levels are typically higher in GSD types III, VI, and IX, and increased levels tend to persist despite treatment [31]. In a study involving 21 patients diagnosed with GSD type III, the median values of ALT and AST were found to be above the laboratory reference values. However, while these values were higher in patients who progressed to cirrhosis, no statistical difference was detected [27]. In a study examining patients with GSD type VI, elevated AST/ALT levels were observed in 93% of the patients, and it was noted that TG levels were mildly elevated in these patients [32]. In a study evaluating the subtypes of GSD type IX, it was reported that patients with GSD type IXc had higher levels of AST/ALT and hypertriglyceridemia compared to patients with GSD types IXa and IXb [28]. In a study conducted by Lu et al. on children diagnosed with GSD type VI in China, it was reported that transaminase elevation occurred in 10 patients (91%), while hyperlipidemia was observed in 3 patients (27%) [33]. In the present study, the mean ALT and AST levels were above the reference values for all GSD types. Mean AST and ALT levels were highest in GSD types III and IXc and lowest in types 0, IXb, and Ib. In our patients, AST and ALT levels tended to remain high despite treatment in GSD types III, IV, and IXc. The reason for this may be the more permanent liver damage observed in types IV and IXc, while in type III, muscle involvement could be a contributing factor. When we looked at our patients, 95.5% had elevated AST and 84.8% had elevated ALT levels at the time of diagnosis, reinforcing the conclusion that elevated levels of these tests are a significant indicator of GSD. Furthermore, it is noteworthy that in 50% of type Ib and type IXb patients, ALT levels are normal even when AST levels are elevated, which can be a significant clue for diagnosing these types. Additionally, the normalization of AST and ALT levels during follow-up, especially in type Ib and type IXb patients, is an important finding.

In a retrospective multicenter study, it was reported that hypercholesterolemia and hypertriglyceridemia were more prevalent and severe in patients with GSD type Ia compared to patients with GSD type Ib. Hypercholesterolemia was reported in 53.4% of patients with GSD type Ia and 14% of patients with GSD type Ib, while these rates were 91.3% and 78.6% for hypertriglyceridemia, respectively. The same study reported that 57% of the patients had uric acid levels high enough to require xanthine oxidase (XO) inhibitors. Among the patients using XO inhibitors, 29% continued to have elevated serum uric acid concentrations despite treatment, and 14% experienced complications related to hyperuricemia [10]. In the present study, TG levels were found to be particularly high in GSD type Ia and type Ib patients compared to other groups. The finding of higher TG levels in GSD type Ia and type Ib patients compared to other groups aligns with other studies in the literature. In our patients, 12.9% had elevated uric acid levels requiring treatment, with nephrolithiasis developing as a complication in four cases. Similarly, when the AST, ALT, and TG levels of the 13 patients who reached adulthood were evaluated before and after, it was observed that the final values decreased compared to the initial values; however, this decrease was not statistically significant, except for the ALT levels. In patients aged > 18 years, the mean ALT values were 232.4 ± 192.6 U/L at the time of diagnosis and 103.1 ± 53.6 U/L at the final follow-up (*P* < 0.05).

Ultrasonographic imaging plays an important role in the evaluation of hepatic GSDs. In GSD type 0, hepatomegaly is not observed due to the deficiency in glycogen synthesis in the liver. Additionally, ketotic hypoglycemia and postprandial hyperglycemia without hyperlacticacidemia and hyperalaninemia during fasting have been reported in the literature [34]. In our study, ketotic hypoglycemia was detected in the only patient with GSD type 0 who presented due to a seizure. The patient was short in stature. There was no hepatomegaly, and liver enzymes were normal. Consistent with the literature, postprandial hyperglycemia was detected during follow-up. Hepatomegaly can be considered a common feature of GSD types I, III, IV, VI, and IX. In a previous study, ultrasonographic findings of patients with GSD type I, diagnosed through liver biopsy, were examined. Hepatomegaly was detected in 13 patients (93%), and increased liver echogenicity was found in 11 patients (79%) [35]. In a study evaluating patients with GSD types VI and IX, hepatomegaly of varying severity, from mild to marked, was observed in the entire patient population, and increased liver echogenicity was frequently noted [32]. In a study evaluating patients with GSD type VI, hepatomegaly was detected in 61 out of 63 patients (96%), and splenomegaly was found in 3 patients (4.8%) [21]. In a study evaluating patients with GSD type IX, hepatomegaly was reported in 93% of patients with type IXa, 94% with type IXb, and 100% with type IXc [28]. Similar to the present study, a previous study reported hepatomegaly at a rate of 80% in patients with hepatic GSDs [36]. In the present study, hepatomegaly was detected in 124 patients (93.9%), and hepatosplenomegaly was found in 27 patients (20.5%), which was consistent with the literature. Among the patients with hepatosplenomegaly, there were 22 with type III, 2 with type Ib, and 1 each with types IV and IXa–c. The age at diagnosis and the disease duration are also important factors influencing the frequency of hepatomegaly. Early diagnosis and initiation of treatment can reduce the development of complications such as hepatomegaly.

In a study evaluating 288 patients with GSD types Ia and Ib, prematurity was reported in 3%, low birth weight in 10%, and congenital heart anomalies in 3% of the cases. Additionally, pancreatitis was reported in three patients and cholelithiasis in two patients [10]. In the literature, a horseshoe kidney was reported in a patient with GSD type Ib [37]. Among the patients included in the present study, the rate of low birth weight was 12.1% (16/132). When examined by type, the rates of low birth weight were as follows: GSD type Ia–b 14.3% (5 out of 35), type III 8.9% (5 out of 56), type VI 22.2% (2 out of 9), type IXb 16.7% (1 out of 6), and type IXc 30% (3 out of 10). The high rate of low birth weight in these patients may be related to the relatively high incidence of twin births observed in our cohort, or to the low availability of actively usable glycogen stores during the intrauterine period. However, it is not possible to make a definitive conclusion regarding this matter. Cholelithiasis was present in only two patients, and one patient had pulmonary stenosis as a congenital heart anomaly. Horseshoe kidney was detected in one patient with GSD type IV.

It has been previously reported that liver transplantation in patients with GSD is performed to treat liver failure or cirrhosis and that complications associated with GSD may develop after liver transplantation. In a study evaluating 25 patients who underwent liver transplantation, it was reported that three of the patients had GSD type III, and all underwent transplantation due to liver failure [11]. In a study involving eight patients who underwent liver transplantation, it was reported that five of these patients were children, and all received transplants due to cirrhosis. The patients demonstrated a good prognosis following the procedure [38]. Two of our patients also underwent liver transplantation. However, one pediatric patient with type IXc who underwent liver transplantation for cirrhosis died due to complications related to the transplant. The other patient was an adult type III patient. Even though hepatic symptoms improved after transplantation, muscle symptoms progressed.

Patients with hepatic GSD may present with similar clinical findings, such as abdominal distension, hepatomegaly, and elevated AST/ALT/TG levels. While the frequently encountered subtypes vary by region, it has been reported that types III and Ia are the most common in Turkey. The findings presented by the Çukurova University Faculty of Medicine Department of Pediatric Metabolism and Nutrition in the differential diagnosis of GSDs are as follows: In the presence of severe hypoglycemia, GSD types Ia and Ib should be considered; if elevated AST levels are not accompanied by elevated ALT levels, GSD types 0, IXb, and Ib should be considered; and if AST/ALT levels are very high, GSD types III and IXc should be considered. Additionally, severe hypertriglyceridemia in GSD types Ia and Ib, neutropenia in type Ib, and elevated uric acid levels in types Ib and III (except type Ia) were identified as indicative findings in subtype differentiation. Although short stature is a common finding in the early stages, it was observed that most patients who can maintain regular follow-up and treatment reach final heights comparable to the family average.

In the present study, it was noteworthy that the frequency of GSD type Ib was higher compared to type Ia. Additionally, the highest reported number of twin patients in the literature was another notable finding of the study. Intestinal duplication cysts, renal artery stenosis, and pulmonary stenosis were structural anomalies associated with GSD that had not been previously reported. However, due to the high frequency of consanguinity within our cohort, these additional pathologies may be incidental. This study highlighted the increased risk of non-hepatic malignancies in patients with GSD type III for the first time. Therefore, in cases with clinical findings primarily indicative of GSD, such as hepatosplenomegaly, but showing a progressive course, non-hepatic malignancies should also be considered.

In hepatic GSDs, which are common and present challenges in differential diagnosis between types and for which there is no curative treatment approach, sharing clinical data from large case series, along with associated anomalies, complications, and the natural course of the disease, will support the diagnosis and follow-up processes.

## Data Availability

The datasets generated and/or analyzed during the current study are not publicly available due to privacy or ethical restrictions.

## Abbreviations

ALL: Acute lymphoblastic leukemia
ALT: Alanine transaminase
AST: Aspartate transaminase
BMI: Body mass index
GSDs: Glycogen storage diseases
SDS: Standard deviation score
TG: Triglyceride
